# Metal *vs.* ligand protonation and the alleged proton-shuttling role of the azadithiolate ligand in catalytic H_2_ formation with FeFe hydrogenase model complexes†
†Electronic supplementary information (ESI) available: Experimental details, UV-vis transient absorption spectra of **1^–^** and **1H**. See DOI: 10.1039/c9sc00876d


**DOI:** 10.1039/c9sc00876d

**Published:** 2019-05-02

**Authors:** Alexander Aster, Shihuai Wang, Mohammad Mirmohades, Charlène Esmieu, Gustav Berggren, Leif Hammarström, Reiner Lomoth

**Affiliations:** a Department of Chemistry-Ångström Laboratory , Uppsala University , Box 523 , SE-751 20 Uppsala , Sweden . Email: reiner.lomoth@kemi.uu.se

## Abstract

Electron and proton transfer reactions of diiron complexes [Fe_2_adt(CO)_6_] (**1**) and [Fe_2_adt(CO)_4_(PMe_3_)_2_] (**4**), with the biomimetic azadithiolate (adt) bridging ligand, have been investigated by real-time IR- and UV-vis-spectroscopic observation to elucidate the role of the adt-N as a potential proton shuttle in catalytic H_2_ formation. Protonation of the one-electron reduced complex, **1^–^**, occurs on the adt-N yielding **1H** and the same species is obtained by one-electron reduction of **1H^+^**. The preference for ligand *vs.* metal protonation in the Fe_2_(i,0) state is presumably kinetic but no evidence for tautomerization of **1H** to the hydride **1Hy** was observed. This shows that the adt ligand does not work as a proton relay in the formation of hydride intermediates in the reduced catalyst. A hydride intermediate **1HHy^+^** is formed only by protonation of **1H** with stronger acid. Adt protonation results in reduction of the catalyst at much less negative potential, but subsequent protonation of the metal centers is not slowed down, as would be expected according to the decrease in basicity. Thus, the adtH^+^ complex retains a high turnover frequency at the lowered overpotential. Instead of proton shuttling, we propose that this gain in catalytic performance compared to the propyldithiolate analogue might be rationalized in terms of lower reorganization energy for hydride formation with bulk acid upon adt protonation.

## Abstract 2

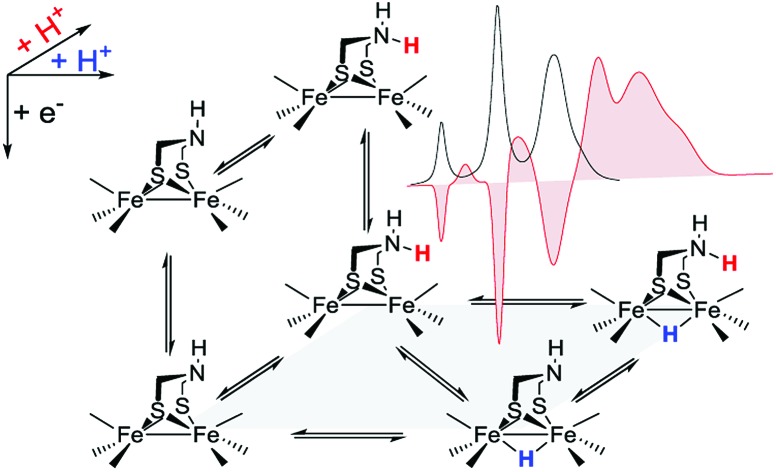
Real-time spectroscopic observation of electron transfer-induced protonation reactivity elucidates the role of the second sphere basic site in a H_2_ evolution catalyst.

## Introduction

Proton binding sites in the second coordination sphere of catalytic metal centres are widely considered as an important design principle for facilitating proton coupled electron transfer in molecular catalysts for *e.g.* water splitting,^1^–^5^ H_2_ formation and activation^6^–^12^ or CO_2_ reduction.^13^–^21^ For the directed design of synthetic catalysts it is, however, important to note that improvements to catalytic performance (TOF, overpotential) brought about by basic sites in the second coordination sphere are not necessarily arising from a proton relay activity. Instead, the modulation of redox and acid/base properties of the metal centre upon changes in protonation state in the second coordination sphere may be the actual origin of improved catalytic performance.^14^–^17^

**Scheme 1:**
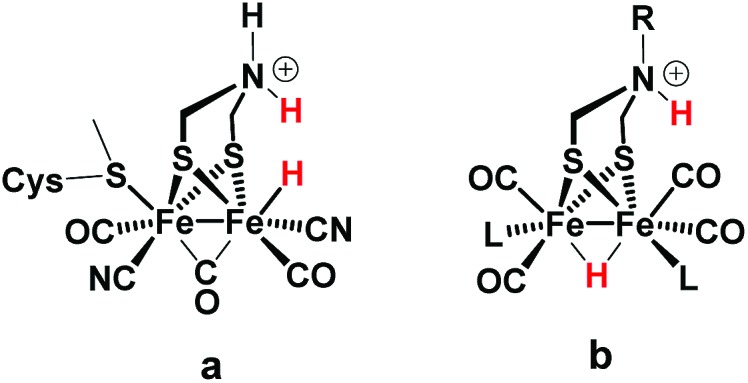
FeFe Hase active site (a) and synthetic model (b) bearing a hydride and a proton.

Regarding catalysts for H_2_ formation, the N_2_P_2_ ligands introduced by DuBois and co-workers,^6^,^7^,^12^ and the azaditiolate (adt) bridging ligand in diiron complexes of the general formula Fe_2_adt(CO)_4_X_2_ (X = *e.g.* CO, R_3_P) modelled after the FeFe-Hase active site,^9^,^22^–^24^ are prominent examples of ligand motifs with basic sites in the second coordination sphere. The amine function of the adt ligand is believed to assist enzymatic H_2_ formation and activation by shuttling of protons to or from the distal iron centre of the active site where the crucial terminal hydride intermediate is formed (Scheme 1a).^25^,^26^ A similar role of the adt ligand in catalysis by the synthetic models considered here seems less likely given the preferred bridging coordination of the hydride (Scheme 1b).^23^ The superior catalytic performance of Fe_2_(adt)(CO)_6_, **1**, over its propyldithiolate (pdt) analogue **2** in electrochemical H_2_ formation has however been attributed to proton shuttling, involving specifically terminal protonation of the metal centre by tautomerization of a adt-NH^+^ precursor upon one-electron reduction of the catalyst.^27^ We were therefore intrigued whether the proposed role of the adt ligand as proton shuttle and the formation of terminal hydride intermediates in these model complexes could be inferred from direct spectroscopic observation. For this purpose we have combined laser flash induced reduction as well as rapid chemical reduction of the catalyst with both UV-vis and IR detection to elucidate structure and reactivity of reduced and reduced-protonated intermediates derived from **1** (ref. 28) and Fe_2_(adt)(CO)_4_(PMe_3_)_2_ (**4**).^29^ This approach enabled us to exclude any proton shuttling role of the adt bridging ligand in catalytic H_2_ formation while the kinetics of ligand and metal protonation steps suggest an alternative rationale for the improved performance.
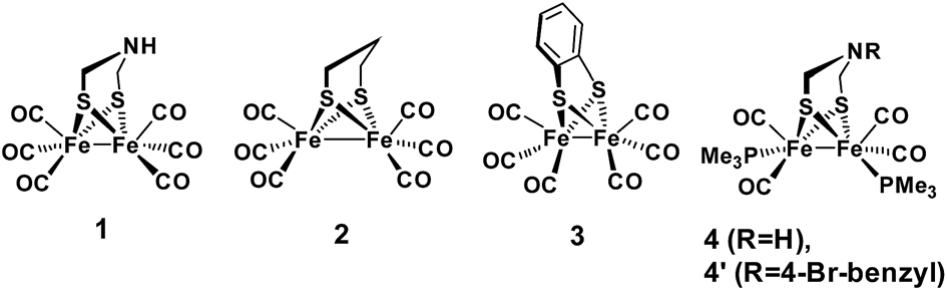


## Results and discussion

**Scheme 2:**
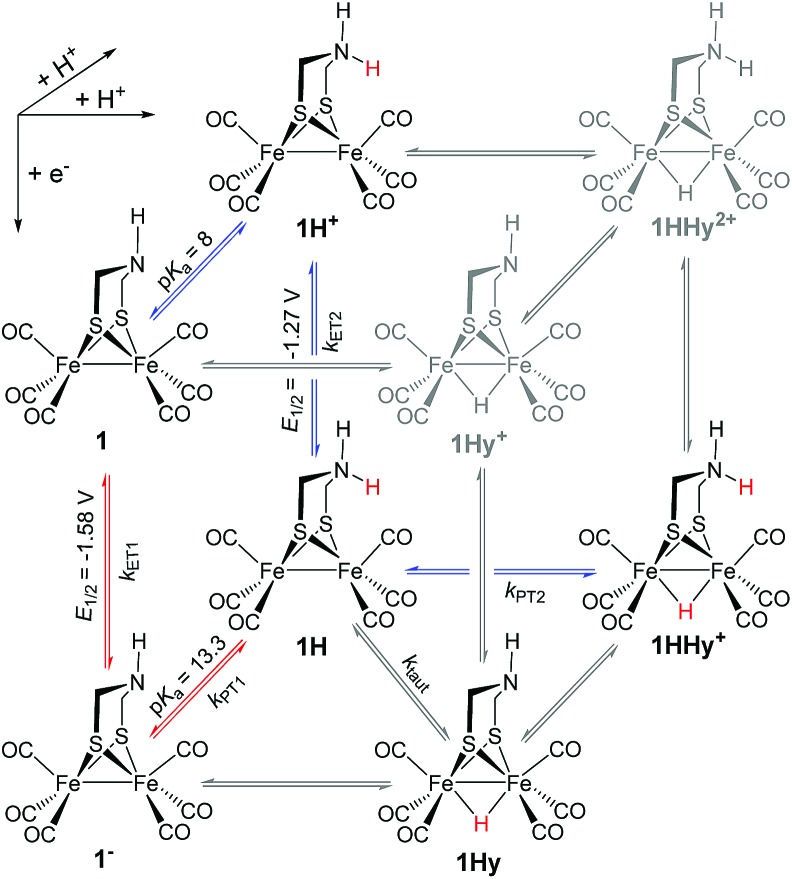
Metal and ligand protonation reactions of **1** (Fe_2_(i,i)) and **1^–^** (Fe_2_(i,0)).

Generally, complex **1** catalyses H_2_ formation *via* an initial ET-PT sequence with weaker acids, or a PT-ET sequence when stronger acids are employed that initially protonate the adt-N with a p*K*_a_ of 8 in acetonitrile^30^ (Scheme 2, potentials from ref. 27).

### Reduction of **1**

**Fig. 1:**
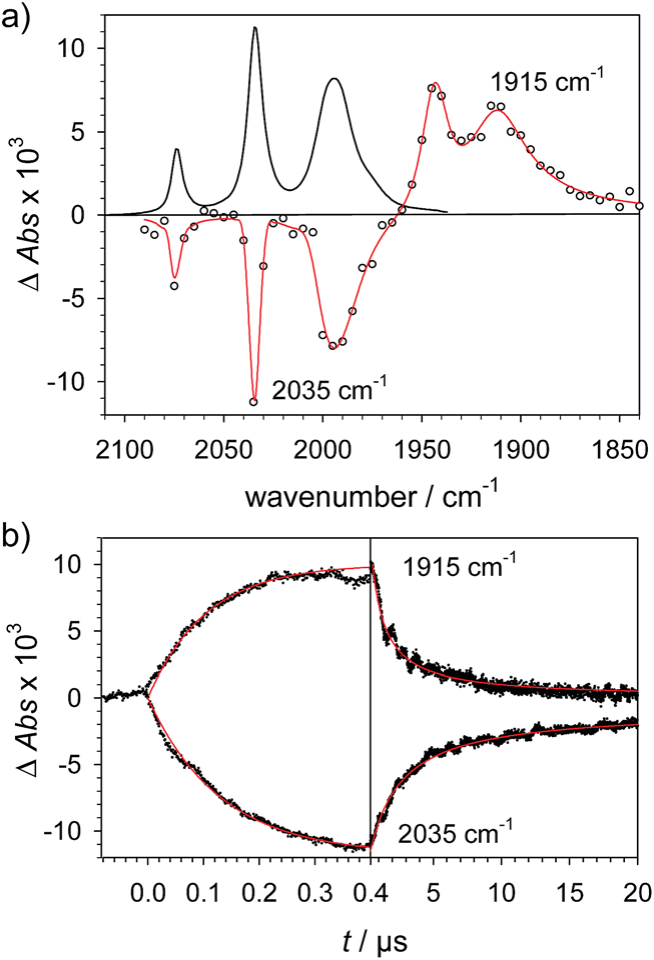
(a) Transient IR spectrum 1 μs after excitation (○, 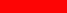) and normalized IR spectrum of the starting state ( <svg xmlns="http://www.w3.org/2000/svg" version="1.0" width="16.000000pt" height="16.000000pt" viewBox="0 0 16.000000 16.000000" preserveAspectRatio="xMidYMid meet"><metadata> Created by potrace 1.16, written by Peter Selinger 2001-2019 </metadata><g transform="translate(1.000000,15.000000) scale(0.005147,-0.005147)" fill="currentColor" stroke="none"><path d="M0 1520 l0 -160 1360 0 1360 0 0 160 0 160 -1360 0 -1360 0 0 -160z"/></g></svg> ) for the reduction of **1** (1.3 mM) by flash-quench generated [Ru(dmb)_3_]^+^ in acetonitrile. (b) Kinetic traces (···) and fits (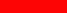) monitoring the pseudo-first order formation of **1^–^** (1915 cm^–1^) and bleach of **1** (2035 cm^–1^) followed by second order charge recombination with TTF^+^.

To investigate the ET-PT route, the one-electron reduced catalyst **1^–^** was obtained by rapid electron transfer from laser flash generated [Ru(dmb)_3_]^+^ according to eqn (1) and (2) (*k*_ET1_ = 3 × 10^9^ M^–1^ s^–1^, eqn (2)). The electronic absorption bands of **1^–^** (570, 700 nm, see ESI†) and the carbonyl region of its IR spectrum (1915, 1945, 2005 cm^–1^, Fig. 1a) resemble closely the spectra reported for the singly reduced pdt analogue **2^–^**.^31^,^32^ The 700 nm band and the uniform shift of the three *ν*_C–O_ bands by 80–90 cm^–1^ towards lower wavenumbers are distinct markers of the intact (μ^2^,κ^2^-adt)Fe_2_(CO)_6_ core in the reduced complex **1^–^**, in contrast to the dissociation of a sulphur–iron bond in the reduced bdt analogue (bdt = benzene-1,2-dithiolate) **3^–^**.^33^ In the absence of proton sources, decay of **1^–^** leads to complete recovery of **1** by diffusion controlled charge recombination with the electron donor radical TTF^+^ (*k*_rec,1_ = 6 × 10^10^ M^–1^ s^–1^, eqn (3)).
1

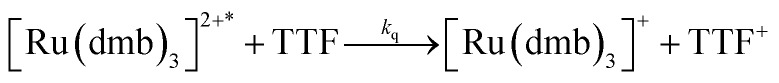



2

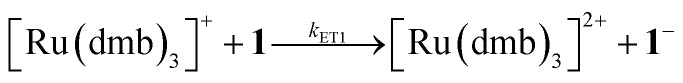



3

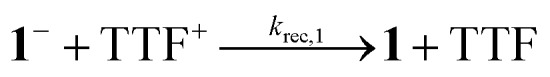




### Metal *vs.* ligand protonation of **1^–^**

**Fig. 2:**
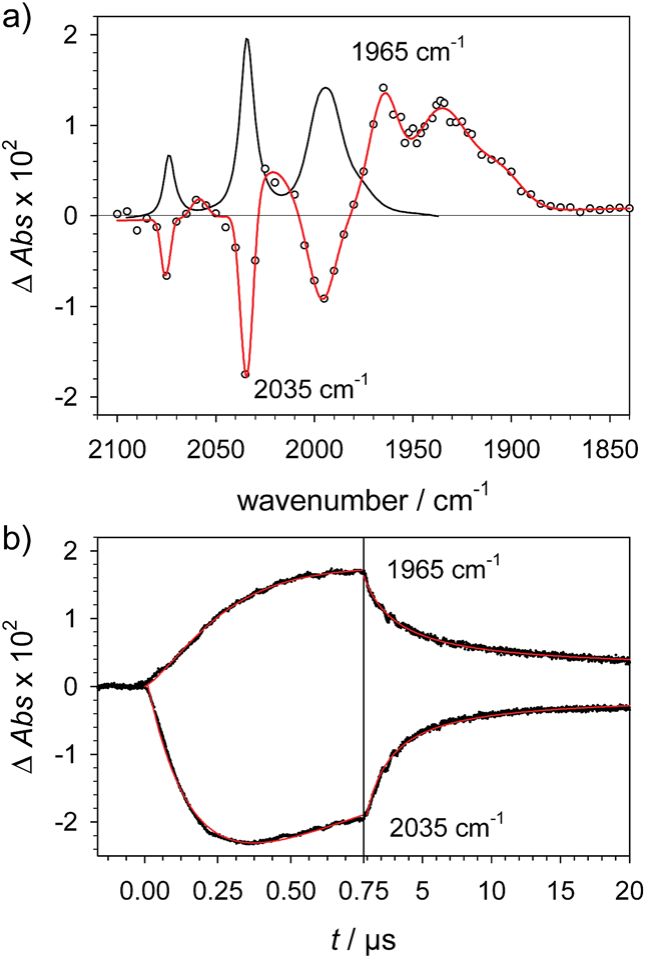
(a) Transient IR spectrum 1 μs after excitation (○, 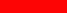) and normalized IR spectrum of the starting state ( <svg xmlns="http://www.w3.org/2000/svg" version="1.0" width="16.000000pt" height="16.000000pt" viewBox="0 0 16.000000 16.000000" preserveAspectRatio="xMidYMid meet"><metadata> Created by potrace 1.16, written by Peter Selinger 2001-2019 </metadata><g transform="translate(1.000000,15.000000) scale(0.005147,-0.005147)" fill="currentColor" stroke="none"><path d="M0 1520 l0 -160 1360 0 1360 0 0 160 0 160 -1360 0 -1360 0 0 -160z"/></g></svg> ) for formation of **1H** by protonation of **1^–^** with Cl_3_CCOOH (13 mM) following reduction of **1** (1.3 mM) by flash-quench generated [Ru(dmb)_3_]^+^ in acetonitrile. (b) Kinetic traces (···) and fits (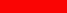) monitoring pseudo-first order formation of **1H** (1965 cm^–1^) and bleach of **1** (2035 cm^–1^) followed by second order charge recombination with TTF^+^.

**Fig. 3:**
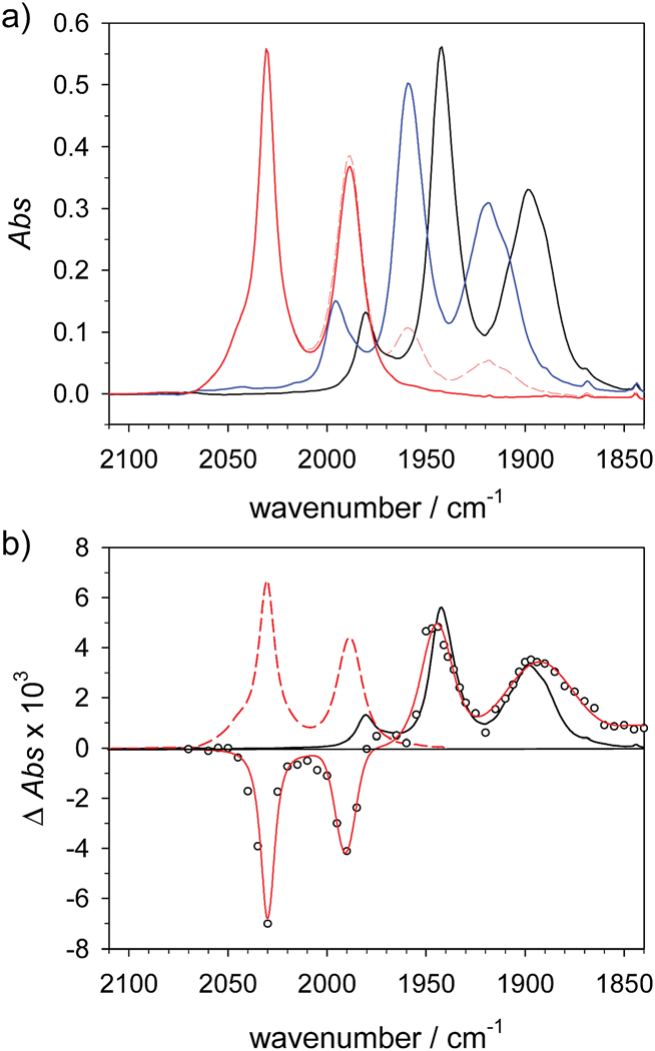
(a) FTIR spectra of **4** (1.5 mM) ( <svg xmlns="http://www.w3.org/2000/svg" version="1.0" width="16.000000pt" height="16.000000pt" viewBox="0 0 16.000000 16.000000" preserveAspectRatio="xMidYMid meet"><metadata> Created by potrace 1.16, written by Peter Selinger 2001-2019 </metadata><g transform="translate(1.000000,15.000000) scale(0.005147,-0.005147)" fill="currentColor" stroke="none"><path d="M0 1520 l0 -160 1360 0 1360 0 0 160 0 160 -1360 0 -1360 0 0 -160z"/></g></svg> ) in acetonitrile, **4H^+^** (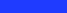) obtained by protonation with toluenesulfonic acid (3 mM), and **4Hy^+^** (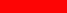) obtained by protonation with toluenesulfonic acid (3 mM) in presence of tetrabutyl-ammonium chloride (1.5 mM) after correction of the raw spectrum (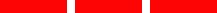) for minor contribution from **4H^+^**. (b) Transient IR spectrum upon reduction of **4Hy^+^** to **4Hy** by electron transfer from flash-quench generated [Ru(dmb)_3_]^+^ (○, 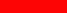, 1 μs after excitation) and normalized IR spectra of **4Hy^+^** (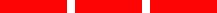) and **4** ( <svg xmlns="http://www.w3.org/2000/svg" version="1.0" width="16.000000pt" height="16.000000pt" viewBox="0 0 16.000000 16.000000" preserveAspectRatio="xMidYMid meet"><metadata> Created by potrace 1.16, written by Peter Selinger 2001-2019 </metadata><g transform="translate(1.000000,15.000000) scale(0.005147,-0.005147)" fill="currentColor" stroke="none"><path d="M0 1520 l0 -160 1360 0 1360 0 0 160 0 160 -1360 0 -1360 0 0 -160z"/></g></svg> ).

Protonation of the laser flash generated **1^–^** (eqn (4)) with weak acids (Cl_3_CCOOH, p*K*_a_ = 10.6 or ClCH_2_COOH, p*K*_a_ = 15.3),^34^ which avoids protonation of the neutral parent complex, shifts all three *ν*_C–O_ bands by about 20 cm^–1^ to higher wavenumbers (2025, 1965 and 1935 cm^–1^), as shown in Fig. 2. The magnitude of the shift is similar to what is observed upon protonation of the neutral parent complex with stronger acids (*cf.*Fig. 3a) and characteristic for protonation of the adt-N.^29^
4

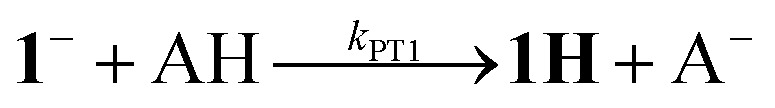




Metal protonation of **1^–^** is on the other hand expected to shift the carbonyl bands by about 80 cm^–1^ to higher wavenumbers. Shifts of this magnitude have been established for the bridging hydrides formed by protonation of Fe_2_(i,i) complexes with electron donor ligands like **4′** (ref. 35) as well as transiently generated Fe_2_(i,0) hexacarbonyl complexes **2^–^** and **3^–^**.^32^,^33^ Mutually cancelling shifts of the carbonyl bands upon one-electron reduction and metal protonation were further evidenced with help of complex **4** (*vide infra*). The transient IR spectrum of the protonation product hence proves that the adt ligand remains the preferred site of protonation, at least kinetically, also upon one-electron reduction of **1**. This conclusion is corroborated by the UV-vis spectrum of **1H** (ESI†), which is similar to its precursor **1^–^**, while metal protonation of **2^–^** was previously shown to cause complete bleach of its visible absorption bands that arise from transitions involving predominantly metal-based orbitals. The observed pseudo-first order rate constants for formation of **1H** follow a linear dependence on acid concentration before becoming limited by the preceding electron transfer step (ESI†). The straightforward concentration dependence excludes any complications of the protonation kinetics due to dimerization of the acetic acids that was previously found to result in quadratic concentration dependence corresponding to an increase in effective acid strength.^36^ Interestingly, protonation of **1^–^**, which occurs unambiguously at the adt-N, proceeds remarkably slowly with bimolecular rate constants *k*_PT1_ of 4 × 10^8^ M^–1^ s^–1^ and 2 × 10^7^ M^–1^ s^–1^ for Cl_3_CCOOH and ClH_2_CCOOH, respectively.

Notably, the protonation rate constant is almost two orders of magnitude below the diffusion controlled limit even for the strongly exergonic reaction with Cl_3_CCOOH (Δp*K*_a_ = 3). This is quite untypical for protonation of an amine base and indicative of significant reorganization associated with ligand protonation.

### Spectral comparison against the one-electron reduced hydride **4Hy**

The above assignment of the protonation reaction to ligand protonation builds to a large extent on the general notion of nearly perfectly cancelling shifts in carbonyl frequencies caused by one-electron reduction and formation of a bridging hydride. This expectation could previously only be supported by the vanishing transient absorption observed upon protonation of complexes **2^–^** and **3^–^**.^32^,^33^ Here, these assignments could be eventually verified by the positive observation of a one-electron reduced hydride **4Hy** by laser flash induced reduction of hydride complex **4Hy^+^**. The latter can be obtained by the Cl^–^-catalysed protonation of **4** that yields the thermodynamically stable hydride instead of the kinetically preferred ligand protonated **4H^+^** in analogy to the previously reported behaviour of **4′**.^37^ Upon one electron reduction of **4Hy^+^** the carbonyl bands of the product **4Hy** coincide almost perfectly with those of the parent complex **4** (Fig. 3) verifying the close spectral resemblance of the diiron carbonyl complexes and their bridging hydride derivatives in the Fe_2_(i,0) oxidation state.

### Probing possible tautomerization of **1H**

According to the above arguments, also formation of a possibly more stable hydride **1Hy** by tautomerization (5) should lead to complete decay of all transient absorption given the predictable spectral similarity between **1** and **1Hy**.
5

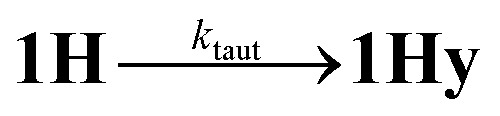




While the expected spectral changes are the same as for charge recombination between **1H** and TTF^+^(6), tautomerization should nevertheless accelerate the decay of **1H** if it proceeds with a rate that is at least comparable to charge recombination (6).
6

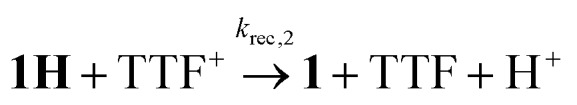




In flash photolysis experiments, **1H** decays with second order kinetics on a time scale of about 10 μs in transient IR and about 100 μs with the lower transient concentrations in UV-vis experiments. These time scales are typical for diffusion controlled recombination (*k*_rec,2_ = 1.5 × 10^10^ M^–1^ s^–1^) and put an upper limit on the order of 10^4^ s^–1^ on the rate constant of a possibly competing tautomerization reaction.

**Fig. 4:**
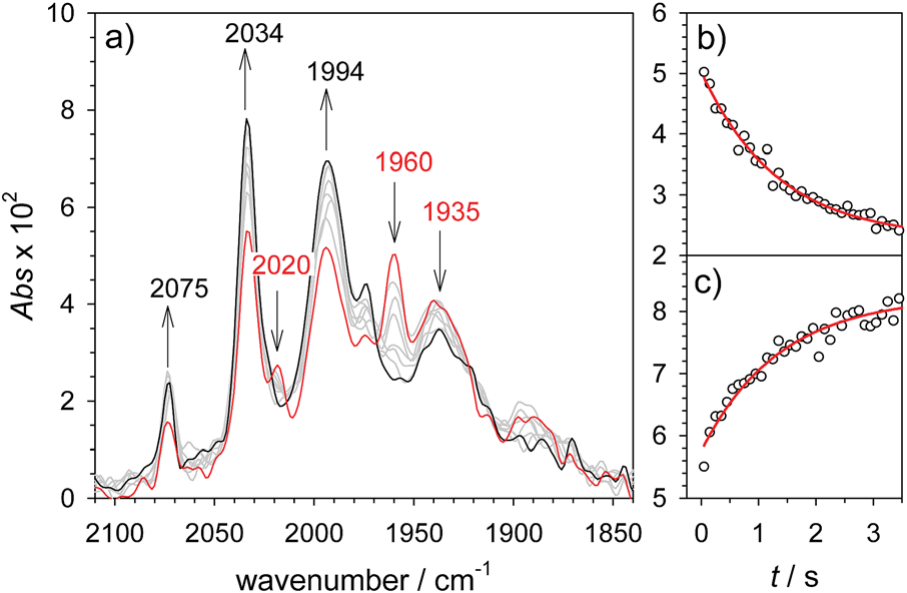
Rapid scan FTIR spectra in acetonitrile between 50 ms (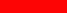) and 3 s ( <svg xmlns="http://www.w3.org/2000/svg" version="1.0" width="16.000000pt" height="16.000000pt" viewBox="0 0 16.000000 16.000000" preserveAspectRatio="xMidYMid meet"><metadata> Created by potrace 1.16, written by Peter Selinger 2001-2019 </metadata><g transform="translate(1.000000,15.000000) scale(0.005147,-0.005147)" fill="currentColor" stroke="none"><path d="M0 1520 l0 -160 1360 0 1360 0 0 160 0 160 -1360 0 -1360 0 0 -160z"/></g></svg> ) after after mixing of **1H^+^** (1.3 mM **1**, 2.6 mM Cl_2_BSA) with cobaltocene (20 mM) (a) and kinetic traces (○) with exponential fits (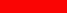) monitoring decay of **1H** at 1960 cm^–1^ (b) and formation of **1** at 2034 cm^–1^ (c).

As a turnover frequency (TOF) of 10^3^ to 10^4^ s^–1^ has been proposed from electrocatalytic experiments (log(*k*_cat_/s^–1^) = 3.9 and 2.6 for 6 mM Cl_3_CCOOH and ClH_2_CCOOH, respectively),^27^ we wanted to probe the reactivity of **1H** on a longer time scale than 100 μs. Thus, **1H^+^** was chemically reduced by cobaltocene in IR stopped flow experiments (rapid-scan FTIR). **1H^+^** was obtained by addition of two equivalents of 2,5-dichlorobenzenesulfonic acid (Cl_2_BSA, p*K*_a_ = 6.7)^34^ to warrant quantitative protonation of **1**. Reduction to **1H** occurred on a shorter time scale than the time resolution of the experiment. Importantly, **1H** was observed for several seconds (Fig. 4). Its decay with a lifetime of 1.3 s yields a product spectrum that can be assigned to the parent complex **1** or the tautomerization product **1Hy** given the expected similarity of their spectra. The former could be generated by catalytic turnover that leads to depletion of acid and therefore regenerates unreduced, unprotonated catalyst. Whether the slow decay of **1H** is predominantly due to turnover or tautomerization cannot be discriminated but the observed lifetime demonstrates in either case that the rate constant for tautomerization, if this occurs at all, has to be smaller than 1 s^–1^. We note that tautomerization would be an intramolecular process that is independent on external conditions, such as identity and concentration of acid and the method of reduction (chemical or electrochemical).

### Reduction and subsequent protonation of **1H^+^**

Alternative formation of **1H***via* the PT-ET route was studied by laser-flash induced reduction of **1H^+^**(7).
7

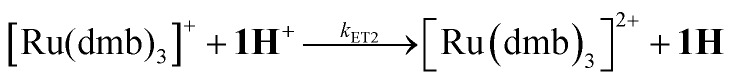




**Fig. 5:**
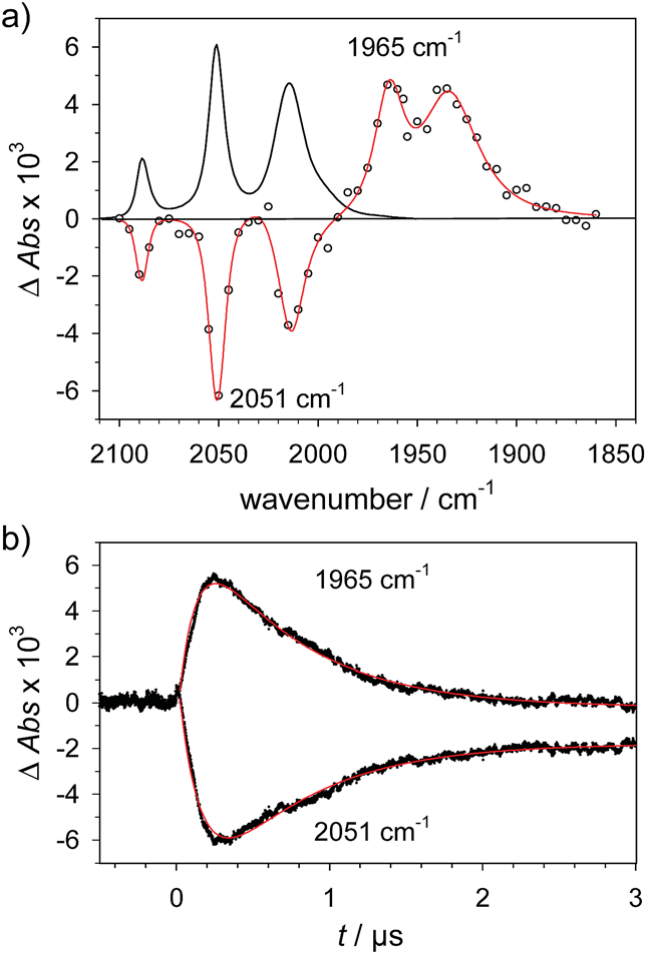
(a) Transient IR spectrum 0.3 μs after excitation (○, 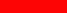) and normalized IR spectrum of the starting state ( <svg xmlns="http://www.w3.org/2000/svg" version="1.0" width="16.000000pt" height="16.000000pt" viewBox="0 0 16.000000 16.000000" preserveAspectRatio="xMidYMid meet"><metadata> Created by potrace 1.16, written by Peter Selinger 2001-2019 </metadata><g transform="translate(1.000000,15.000000) scale(0.005147,-0.005147)" fill="currentColor" stroke="none"><path d="M0 1520 l0 -160 1360 0 1360 0 0 160 0 160 -1360 0 -1360 0 0 -160z"/></g></svg> ) for formation of **1H** by reduction of **1H^+^** (obtained by protonation of 1 with 2.6 mM Cl_2_BSA) with flash-quench generated [Ru(dmb)_3_]^+^ in acetonitrile. (b) Kinetic traces (···) and fits (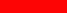) monitoring pseudo-first order formation of **1H** (1965 cm^–1^) and bleach of **1H^+^** (2051 cm^–1^) followed by pseudo-first order decay of **1H** by protonation with Cl_2_BSA and charge recombination with TTF^+^.

**1H^+^** was obtained by protonation of **1** with Cl_2_BSA that shifts the IR peaks by about 20 cm^–1^ to higher wavenumbers (2090, 2051 and 2015 cm^–1^).^29^ The transient IR absorption spectrum after reduction of **1H^+^** (Fig. 5) shows the bleaching of all three *ν*_C–O_ peaks of **1H^+^** together with the same product absorption peaks previously attributed to **1H** (2025, 1965, 1935 cm^–1^). While the ET-PT and PT-ET sequences lead expectedly to the same product **1H**, its decay is much faster when generated in presence of the stronger acid required for the initial ligand protonation. The accelerated decay of **1H** (Fig. 5) without appearance of new transient signals can be assigned to its metal protonation yielding **1HHy^+^**(8).
8

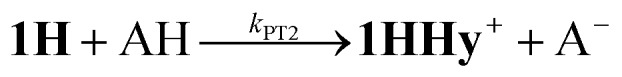




This assignment is based on the expected IR absorption of **1HHy^+^** that is likely to cancel the bleach of absorption from the starting material **1H^+^** as previously found for hydrides **2Hy** and **3Hy**.^32^,^33^ Protonation is also manifested in a slower charge recombination of **1HHy^+^** with TTF^+^ (ESI†). Kinetic analysis of both **1H** and TTF^+^ signals results in a protonation rate constant of *k*_PT2_ = 6 × 10^7^ M^–1^ s^–1^ (see ESI†). The analysis eliminates the possible effect of accelerated recombination with accumulated TTF^+^ in the presence of acid. With a rate constant of 6 × 10^7^ M^–1^ s^–1^ formation of the hydride **1HHy^+^** by metal protonation of **1H** with Cl_2_BSA (p*K*_a_ 6.7)^34^ occurs about as fast as metal protonation of the pdt analogue **2^–^** (7 × 10^7^ M^–1^ s^–1^)^32^ by TsOH (p*K*_a_ 8–8.7).^34^ Both complexes are in the same Fe_2_(i,0) oxidation state but the p*K*_a_ of the hydride should drop by about 5 units according to a shift of +330 mV in reduction potential for the **1H^+^**/**1H** couple compared to **1**/**1^–^** or **2**/**2^–^**. Protonation of **1H**, even by the stronger Cl_2_BSA, is therefore thermodynamically less favourable than protonation of **2^–^** by TsOH, with Δp*K*_a_ lowered by about 3 for the former reaction. Applying the free energy relationship (Brønsted coefficient of 0.4) established for protonation of **2^–^**,^32^ the protonation of **1H** to **1HHy^+^** is hence one to two orders of magnitude faster than expected for protonation of its pdt analogue **2^–^** with the same driving force. Intrinsically faster protonation of the adt-protonated **1H** would prevent this step from becoming rate limiting when reducing weaker acids. Below, we discuss the possible origin of this favourable effect.

### Implications for (electro)catalytic H_2_ formation

From previous electrochemical experiments comparing **1** and **2** it has been concluded that catalytic H_2_ formation from the same acids proceeds with similar second order catalytic rate constants despite the lower overpotential arising from the easier reduction of adt-protonated catalyst.^27^ Undiminished reactivity towards protonation despite the lowered driving force would imply that **1** breaks the usual scaling relation between overpotential and rate,^38^ an effect that has been attributed to the proton shuttling role of the adt^27^ ligand. By putting an upper limit of 1 s^–1^ on the first order rate constant for intramolecular tautomerization, our results exclude however that a mechanism involving hydride formation by proton shuttling at the Fe_2_(0,i) redox level could under any conditions result in the reported pseudo-first order catalytic rate constant on the order of 10^3^ to 10^4^ s^–1^, as previously proposed.^27^ It is important to note that the reported kinetics refer to the first catalytic wave (–1.4 V) that has been attributed to mechanisms where the hydride is formed prior to the second electron transfer, *i.e.* a CECE sequence with stronger acids or an ECCE sequence with weak acids. Therefore, possible formation of the hydride *via* tautomerization at the Fe_2_(0,0) redox level (**1H^–^** → **1Hy^–^**) cannot explain the superior catalytic performance of the adt complex.

An alternative advantage of the adt ligand might instead arise from the lowered barrier for hydride formation due to the structural changes at the iron core induced by the preceding ligand protonation. This notion would be in line with the relative sluggishness of the latter reaction (see above) and is corroborated by structural data that has been previously obtained from EXAFS spectra^39^ of ligand- and metal-protonated states of the related Fe_2_(i,i) complex **4′**.¶
¶Protonation kinetics of the phosphine complexes **4** (ref. 29) and **4′** (ref. 37) have so far been studied only in the Fe_2_(i,i) state where hydride formation is extremely slow.
37 Specifically, protonation of the adt ligand in **4′** results in an elongated Fe–Fe bond distance in **4′H^+^** that is close to the distance found in the bridging hydride **4′HHy^2+^**. Formation of the hydride from the ligand protonated complex requires hence much less reorganization of this coordinate than formation of hydride **4′Hy^+^** from **4′**.

Generally, a proton shuttling ligand could accelerate not only the formation of the hydride intermediate but also its subsequent coupling with a proton. In case of adt complex **1**, with its bridging hydride intermediate, neither of the two steps is, however, likely to involve the adt-N, for steric reasons. In contrast to the terminal hydride of the enzyme active site, the hydride is bridging in the model complex (*cf.*Scheme 1), as evidenced by the conserved symmetry shown by the IR spectra, and the absence of a bridging CO IR band. The superior catalytic performance of **1** over its pdt analogue **2** is instead attributed to the observed acceleration of hydride formation with bulk acid as described above.

## Conclusions

In summary, we could demonstrate by real-time spectroscopic observation that the initial protonation of hydrogenase model complex **1**, also in its one-electron reduced Fe_2_(i,0) state **1^–^**, occurs on the adt ligand rather than the Fe_2_ core. The same ligand protonated intermediate **1H** was alternatively generated by one-electron reduction of **1H^+^** and no evidence for tautomerization of **1H** to a potentially more stable hydride **1Hy** was observed in either case. **1H** was stable on the time scale of 1 s, which shows that intramolecular proton shuttling cannot be responsible for the reported catalytic TOFs of 10^3^ to 10^4^ s^–1^.^27^ For catalytic H_2_ generation from weak acids this implies that no hydride intermediate is formed on the Fe_2_(i,0) level; instead, hydride formation may occur after further reduction, by direct protonation in the Fe_2_(0,0) state. Only with strong acids a doubly protonated intermediate **1HHy^+^** is formed in the Fe_2_(i,0) state, by direct metal protonation of **1H**, not by ligand protonation of a hydride precursor. Generally, it can therefore be concluded that, in contrast to the enzymatic reaction, the adt ligand of model complex **1** has no proton relay function that enables rapid formation of the bridging hydride intermediates on the Fe_2_(i,0) level. The protonation kinetics reveal however an exceptionally high barrier for protonation of the adt ligand that we suggest in turn may lower the barrier for hydride formation, presumably due to rearrangements of the metal core upon ligand protonation. In this way the basic site in the second coordination sphere might allow the catalyst to overcome the usual trade-off between overpotential and rate without invoking any proton-shuttling role.

## Conflicts of interest

There are no conflicts to declare.

## Supplementary Material

Supplementary information
