# Supplementary material for: Metal vs. ligand protonation and the alleged proton-shuttling role of the azadithiolate ligand in catalytic H2 formation with FeFe hydrogenase model complexes
Source: Chem Sci. 2019 May 2;10(21):5582–8. doi: 10.1039/c9sc00876d (PMC6552503; doi:10.1039/c9sc00876d)
Supplement: Supplementary file 1 [file SC-010-C9SC00876D-s001.pdf]

Electronic Supplementary Material (ESI) for Chemical Science.
This journal is © The Royal Society of Chemistry 2019

Supporting information

Metal vs. ligand protonation and the alleged proton-shuttling role of the azadithiolate ligand in catalytic H_2 formation with FeFe hydrogenase model complexes

Alexander Aster, Shihuai Wang, Mohammad Mirmohades, Charlène Esmieu, Gustav Berggren, Leif Hammarström and Reiner Lomoth

Contents

1. UV-Vis transient absorption spectra of 1^- and 1H 2
2. Protonation kinetics of 1^- 3
3. Protonation kinetics of 1H........ 3
4. Compilation of IR data 4
5. Experimental 5

1. UV-Vis transient absorption spectra of 1^- and 1H

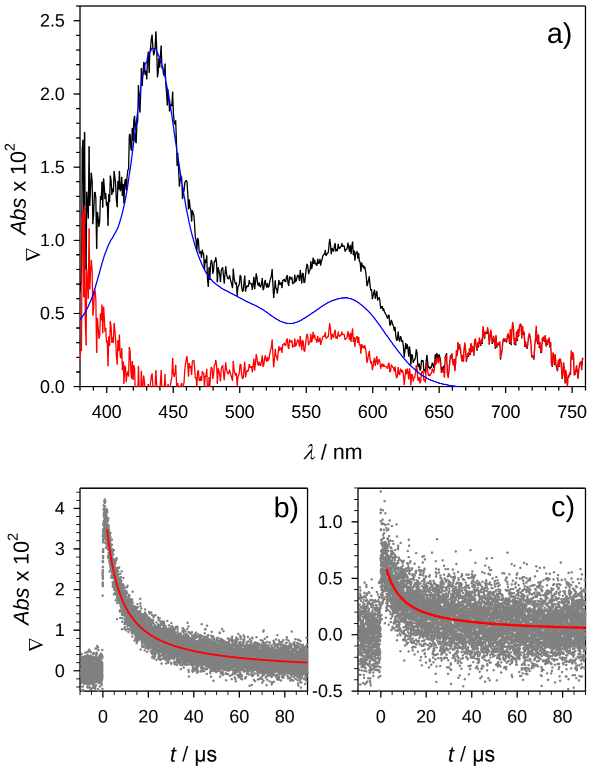

Figure S-1. Reduction of **1** by flash-generated $[Ru(dmb)_3]^+$ (40 µM $[Ru(dmb)_3]^{2+}$, 1.6 mM TTF, 5.9 mM **1**, excitation: 532 nm, 10 mJ/pulse). Transient absorption spectrum (▬) at a delay time of 5 µs and differential spectrum for reduction of **1** to $\mathbf{1}^-$ (▬) obtained by subtraction of spectroelectrochemically determined absorption changes for oxidation of TTF (▬) (a). Kinetic traces (•••) with second order fits (▬) monitoring recombination of TTF^+ at 430 nm (b) and $\mathbf{1}^-$ at 710 nm (c).

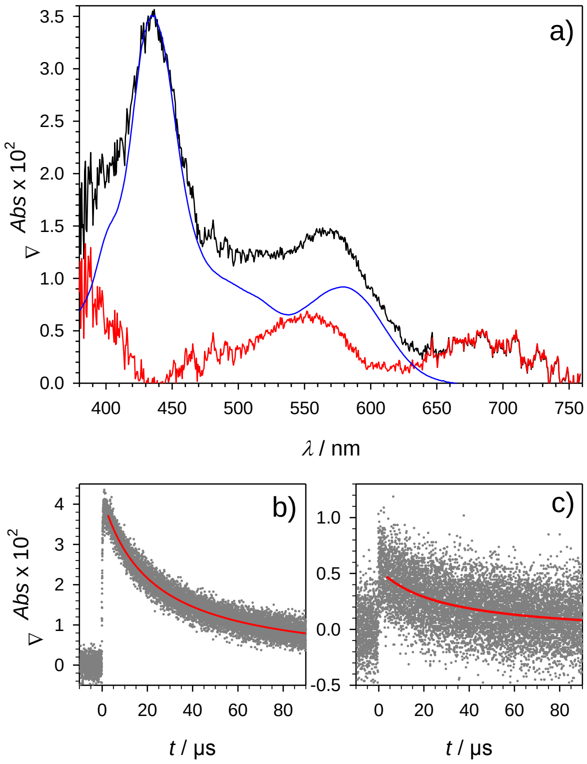

Figure S-2. Reduction of **1** by flash-generated $[Ru(dmb)_3]^+$ and protonation of $\mathbf{1}^-$ by Cl_3CCOOH (40 µM $[Ru(dmb)_3]^{2+}$, 1.6 mM TTF, 5.9 mM **1**, 5.9 mM Cl_3CCOOH, excitation: 532 nm, 10 mJ/pulse). Transient absorption spectrum (▬) at a delay time of 5 µs and differential spectrum for reduction and protnation of **1** to **1H** (▬) obtained by subtraction of spectroelectrochemically determined absorption changes for oxidation of TTF (▬) (a). Kinetic traces (•••) with second order fits (▬) monitoring recombination of TTF^+ at 430 nm (b) and **1H** at 710 nm (c).

2. Protonation kinetics of 1^{-}

Pseudo-first order rate constants of the protonation reaction (4) with different concentrations of Cl_3CCOOH and ClH_2CCOOH were obtained from fits to the rise of IR transient absorption traces monitoring formation of **1H** at 1965 cm^{-1} (cf. Figure 1b). Pseudo-first order rate constants up to about 6×10^{6} s^{-1} are proportional to acid concentration while the preceding electron transfer step (2) becomes rate limiting at higher concentrations of Cl_3CCOOH.

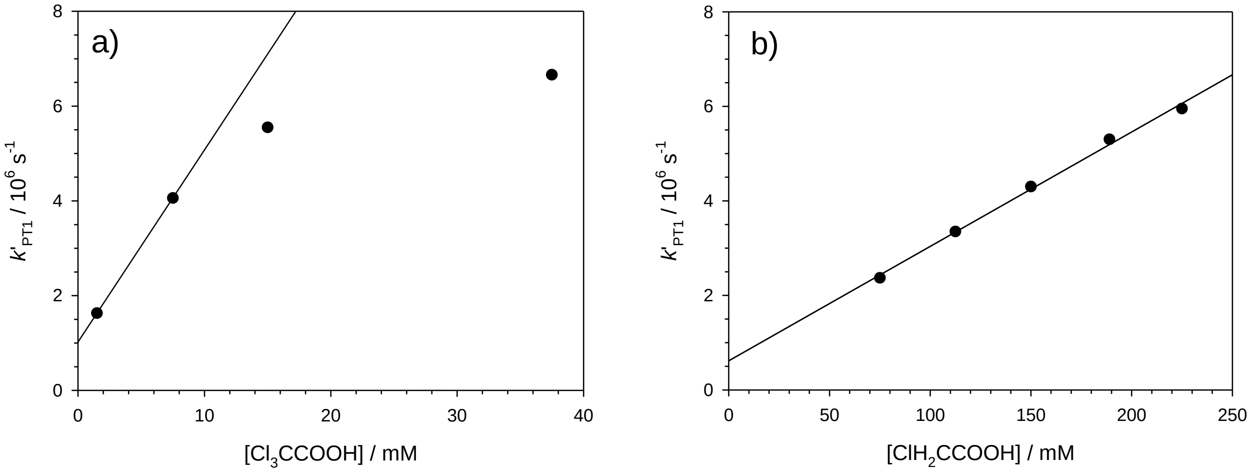

Figure S-3. Pseudo-first order rate constants for the formation of **1H** monitored at 1965 cm^{-1} as a function of acid concentration.

3. Protonation kinetics of 1H

In contrast to protonation of the adt ligand (cf. Figures S-1, S-2), protonation of the metal centres can be expected to bleach the visible absorption bands of **1H** by analogy to the reported spectra for protonation of $\mathbf{2}^{-}$.[1] The kinetics of the reaction between **1H** and Cl_2BSA was therefore also studied by UV-Vis transient absorption spectroscopy. Figure S-4a compares kinetic traces monitoring the decay of **1H** at 710 nm in presence of Cl_3CCOOH (•••, ▬) and in presence of Cl_2BSA (•••, ▬). In presence of Cl_3CCOOH, the second order decay of **1H** can be attributed to charge recombination with TTF^{+} resulting in a corresponding second order decay of the 466 nm absorption of the donor radical (Figure S-4b, •••, ▬). In presence of Cl_2BSA, the decay of **1H** follows faster pseudo-first order kinetics (Figure S-6a, •••, ▬) due to the additional reaction of **1H** with excess of Cl_2BSA. This reaction yields a product that recombines much slower with TTF^{+} than **1H** as evident from the biphasic kinetics observed at 466 nm (Figure S-4b, •••, ▬). The bleaching of the visible absorption bands and the slower recombination of the product with TTF^{+} support the assignment of this reaction to the formation of $\mathbf{1HHy}^{+}$ as suggested by the transient IR data. The decay of **1H** in presence of Cl_2BSA is hence attributed to a combination of charge recombination (eq. 6) and metal protonation (eq. 8) and the observed rate constant is given by eq. 9 where $k'_{PT2} = k_{PT2}[HA]$ and $k'_{Rec,2} = k_{Rec,2}[TTF^{+}]$. This expression takes into account that TTF^{+} is likely to be present in concentrations exceeding the flash generated concentration in strongly acidic solutions where irreversible processes can lead to its accumulation to varying extent.

As only the flash generated concentration is known from transient absorption, recombination will make an uncertain contribution to the decay of **1H**, precluding reliable determination of the protonation rate constant directly from decay of **1H**. The biphasic kinetics of TTF^{+}, provides however the yield of the protonation product by the relative amplitudes of the fast (A_1) and slow (A_2) phase and the protonation rate constant can be obtained from eq. 10.

$$\mathbf{1H} + TTF^{+} \xrightarrow{k_{Rec,2}} \mathbf{1} + TTF + \mathbf{H}^{+} \quad (6)$$

$$\mathbf{1H} + AH \xrightarrow{k_{PT2}} \mathbf{1HHy}^{+} + A^{-} \quad (8)$$

$$k_{obs} = k'_{PT2} + k'_{Rec,2} \quad (9)$$

$$k'_{\mathrm{PT2}} = k_{obs} \frac{1}{\left(\frac{A_1}{A_2}+1\right)} \quad (10)$$

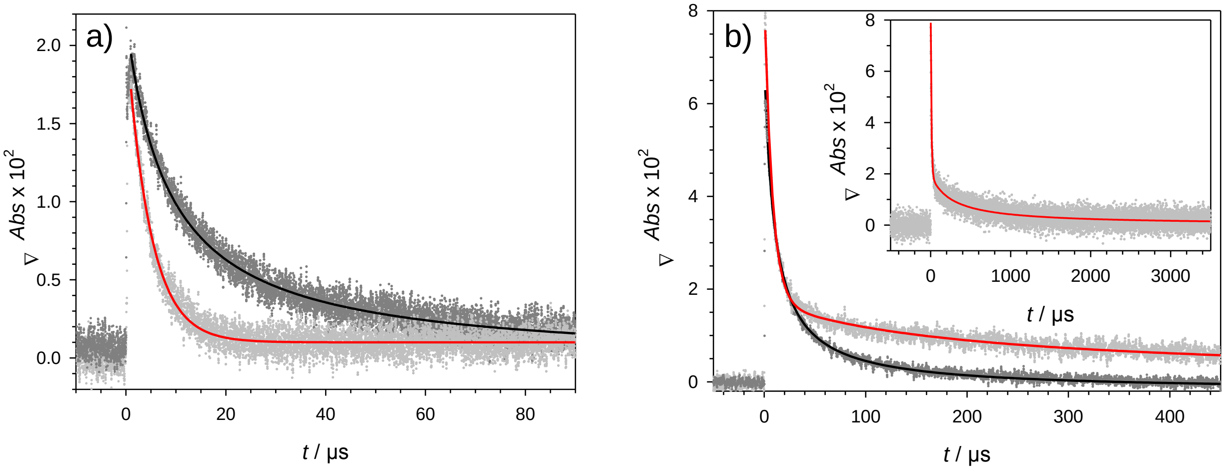

Figure S-4. Decay of transient absorption from **1H** at 710 nm (a) and TTF^+ at 466nm (b). Kinetic traces and fits in presence of 5.3 mM Cl_3CCOOH (•••, ▬) and 0.59 mM Cl_2BSA (•••, ▬). (40 µM $[Ru(dmb)_3]^{2+}$, 1.6 mM TTF, 0.59 mM **1**, with 5.9 mM Cl_3CCOOH or 1.2 mM Cl_2BSA, excitation: 532 nm, 50 mJ/pulse).

Approximating the decay of TTF^+ as a sum of a slow second order decay and a fast pseudo-first order decay, the fit shown in figure S-6b yields $A_1/A_2=3.8$. The kinetics of the fast phase (1.3×10^5 s^{-1}) of TTF^+ decay agree reasonably well with the decay of **1H** (2.0×10^5 s^{-1}). Taking an average of $k_{obs} = 1.7\times10^5$ s^{-1} results in $k'_{PT2} = 3.5\times10^4$ s^{-1}. With the free concentration of Cl_2BSA (5.9×10^{-4} M, half is consumed in the initial formation of **1H**$^+$) a second order rate constant of $k_{PT2} = 6\times10^7$ M^{-1} s^{-1} is obtained for the formation of **1HHy**$^+$.

4. Compilation of IR data

complex	wavenumber/cm^{-1}
**1**	2073, 2034, 1994
**1**$^-$	~2005,[1] 1945, 1915
**1H**$^+$	2090, 2051, 2015
**1H**	~2025,[1] 1965, 1935
**1HHy**$^+$	(2090, 2051, 2015)[2]
**4**	1980, 1942, 1898
**4H**$^+$	1996, 1960, 1921
**4Hy**$^+$	2031, 1990
**4Hy**	~1980,[1] 1944, 1895

[1] Band overlapping with bleach of parent complex. [2] Inferred from cancelling transient absorption upon reduction and protonation of $1H^+$.

5. Experimental

Synthesis

[Fe_2adt$(CO)_6$] (**1**) was prepared according to published procedures.[2] Synthesis of [Fe_2adt$(CO)_4(PMe_3)_2$] (**4**) followed the published procedure[3] with minor modifications. The crude product was purified on a silica column under Ar, eluted with a heptane/DCM solvent gradient (from 4:1 to 1:1). The isolated product was evaporated to dryness under vacuum to yield a deep purple/red solid.

Transient IR and UV-Vis absorption spectroscopy

A frequency doubled Q-switched Nd:YAG laser (Quanta-Ray ProSeries, Spectra-Physics) was employed to obtain 532 nm pump light (FWHM 10 ns, 10 mJ/pulse if not stated otherwise).

IR probe light was provided by two continuous wave quantum cascade (QC) IR lasers with a tuning capability between 1300-1965 cm^{-1} for laser 1 and 1960-2150 cm^{-1} for laser 2 (Daylight Solutions). For IR detection a liquid nitrogen-cooled mercury-cadmium-telluride (MCT) detector (KMPV10-1-J2, Kolmar Technologies, Inc,) connected to a TDS 3052 500 MHz 5 GS/s oscilloscope (Tektronix) was used. The IR probe light was overlapped with the actinic laser beam in a quasi co-linear arrangement at 25° angle. Samples were kept in an "Advanced Liquid Transmission Cell" (Specac, GS20502) with an O-ring (1.5 mm) as spacer between the CaF_2 widows to guarantee oxygen free operation.

UV/Vis probe light was provided by a pulsed XBO 450 W Xenon Arc Lamp (Osram). Transient spectra were recorded with an iStar CCD camera (Andor Technology) of an LP920-S laser flash photolysis spectrometer (Edinburgh Instruments) and single wavelength traces were recorded with an LP920-K PMT detector connected to a TDS 3052 500 MHz 5 GS/s oscilloscope (Tektronix). Measurements were performed at right angle in a fluorescence quartz cuvette (Starna) with 10 × 10 mm pathlength.

All samples were prepared in darkness in an Ar filled Unilab glove box (MBraun), using acetonitrile distilled and degassed by freeze-pump-thaw cycles.

FTIR spectroscopy

FTIR spectra (2 cm^{-1} resolution, 16 averages) were recorded on a Bruker IFS 66v/S FTIR spectrophotometer with OPUS software.

Stopped-flow FTIR spectroscopy

Rapid-mixing transient IR measurements were carried out with a Tgk SFA-20 stopped-flow drive equipped with a flow cell fitted with CaF_2 windows (Tgk Scientific) and a path length of ca. 0.25 mm.

Spectra were collected at 4 cm^{-1} spectral resolution in 50 or 100 ms intervals with the rapid-scanning mode of an IFS 66v/S FT-IR spectrometer (Bruker) equipped with a liquid nitrogen-cooled MCT detector. All solutions were prepared and loaded into gas-tight syringes in a Unilab glove box (MBraun) under argon atmosphere.

(1) Wang, S. H.; Aster, A.; Mirmohades, M.; Lomoth, R.; Hammarström, L. *Inorg. Chem.* **2018**, *57*, 768.

(2) Li, H.; Rauchfuss, T. B. *J. Am. Chem. Soc.* **2002**, *124*, 726.

(3) Wright, J. A.; Webster, L.; Jablonskytė, A.; Woi, P. M.; Ibrahim, S. K.; Pickett, C. J. *Faraday Discuss.* **2011**, *148*, 359.
